# Lipopolysaccharide Stimulated the Migration of NIH3T3 Cells Through a Positive Feedback Between β-Catenin and COX-2

**DOI:** 10.3389/fphar.2018.01487

**Published:** 2018-12-19

**Authors:** Xiao-Jun Li, Feng-Zhen Huang, Yan Wan, Yu-Sang Li, Wei Kevin Zhang, Yang Xi, Gui-Hua Tian, He-Bin Tang

**Affiliations:** ^1^Department of Pharmacology, School of Pharmaceutical Sciences, South-Central University for Nationalities, Wuhan, China; ^2^School of Medicine, Institute of Biochemistry and Molecular Biology, Ningbo University, Ningbo, China; ^3^Dongzhimen Hospital, Beijing University of Chinese Medicine, Beijing, China; ^4^Research Institute of Huazhong University of Science and Technology, Shenzhen, China

**Keywords:** COX-2, β-catenin, scratch assay, fibroblasts migration, NIH3T3 cells

## Abstract

How β-catenin/COX-2 contribute to inflammation-induced fibroblasts migration remains poorly understood. Therefore, in this study, lipopolysaccharide (LPS) was used as a stimulus to accelerate the migration of NIH3T3 cells, which mimicked the tissue repair process. LPS treatment increased the cell migration in concentration-and time-dependent manner. And NS398, a COX-2 inhibitor, inhibited LPS-induced NIH3T3 cells migration. DKK-1, an antagonist of the Wnt/β-catenin signaling, also inhibited that migration. However, TWS119, an inducer of β-catenin via GSK-3β, increased the cell migration. LPS or TWS119 treatment increased COX-2, β-catenin, *TGF-β1*, and *HMGB-1* expressions, and that could be attenuated by NS398 or DKK-1 addition. LPS induced the PGE_2_ production, and PGE_2_ increased the expression and nuclear translocation of β-catenin, while EP2 blocker, AH6809, alleviated those effects. TWS119 increased the luciferase activity in the COX-2 promoter. In conclusion, LPS stimulated the NIH3T3 fibroblasts migration through a positive feedback between β-catenin and COX-2, in which PGE_2_, EP2, *TGF-β1*, and *HMGB-1* played as signal molecules.

## Introduction

Fibroblasts comprise the most abundant cell type in connective tissues and are central players in organ homeostasis. Their main function is to maintain the structural integrity of connective tissue by secreting collagen and fibronectin, which are principal components of the extracellular matrix. Synthesis of new tissue by fibroblasts is required for tissue rebuilding in response to injury. The recruitment of fibroblasts is essential in wound healing but are also central features in pathological mechanisms leading to tissue fibrosis and remodeling (Eming et al., 2017).

Inflammation is part of the complex biological response of body tissues to harmful stimuli. One of the most important function of inflammation is to initiate tissue repair (Pandolfi et al., 2016; Eming et al., 2017). Cyclooxygenase-2 (COX-2) is a key molecule functioning as an early response to pro-inflammatory mediators and stimuli (Rajakariar et al., 2006). In inflammatory status, the COX-2 is rapidly induced, and its activity accounts for the large amounts of prostaglandins seen in inflammation. β-Catenin is another molecule playing important roles in inflammation. Alterations in the localization and expression levels of beta-catenin have been associated with various forms of inflammation. In our previous study, we indicted that there might be a crosstalk between COX-2 and β-catenin in inflammation status (Li et al., 2015). Following a wound, pathways activated by extracellular stimuli including inflammatory factors, have all been shown to be involved in the response in fibroblasts. Along these pathways, β-catenin, and COX-2 often act as intracellular signal molecule, may contribute to inflammation-induced fibroblast migration in tissue repair.

The aforementioned observations lead us to the hypothesis that the crosstalk between COX-2 and β-catenin could be an important regulator of inflammation-induced fibroblasts migration. To clarify that point, fibroblasts migration was induced by lipopolysaccharide (LPS) in NIH3T3 cells with or without selective tools [NS-398, a COX-2 inhibitor; DKK-1, an antagonist of the β-catenin signaling; AH6908, an antagonist of PGE_2_ receptor 2 (EP2)]. The results give us a better interview of the roles of COX-2 and β-catenin in inflammation-induced fibroblasts migration, and might be useful for tissue repair research.

## Materials and Methods

### Cell Culture

NIH3T3 cells were obtained from the China Center for Type Culture Collection (CCTCC, Wuhan, China). The cells were cultured in high-glucose Dulbecco’s modified Eagle’s medium (DMEM) at 37°C in a humidified atmosphere of 5% CO_2_. The media were supplemented with 10% heat-inactivated calf serum and 1% penicillin/streptomycin. The cells were plated on 35-mm dishes or μ-slides with eight wells. LPS from Escherichia coli O111:B4 was purchased from sigma (#L4130). All the cells were passaged 1:4 (35 mm) using 0.25% trypsin when they reached 80–90% confluence. Then, these cells were randomly divided into seven groups: the control group (*n* = 5); the LPS group (400 ng/ml, *n* = 8); the LPS plus NS398 group (LPS, 400 ng/ml; NS398, 10 μM; *n* = 5); the LPS plus DKK-1 group (LPS, 400 ng/ml; DKK-1, 100 ng/ml; *n* = 5); the TWS119 group (10 μM, *n* = 5); and the TWS119 plus NS398 group (TWS119, 10 μM; NS398, 10 μM; *n* = 5); the TWS119 plus DKK-1 group (TWS119, 10 μM; DKK-1, 100 ng/ml; *n* = 5). Recombinant human DKK-1 (Lot # 1110454, purity ≥97%) was purchased from PeproTech.

### Scratch Analysis

The scratch assay was used to evaluate the migration ability of NIH3T3 cells, as described previously (Liang et al., 2007; Felice et al., 2015). Briefly, NIH3T3 cells were grown to full confluence in 35-mm dishes with complete medium and then the cell monolayer was scratched with a sterile pipette tip and washed with medium to remove detached cells from the dishes. Cells were then kept in an incubator with or without treatments of LPS in the fully supplemented cultured medium, which was replaced with Hank’s buffer after 24 h. The wound gap was monitored under a microscope with corresponding images recorded using a digital camera. For each image, distances between one side of scratch and the other were quantified at certain intervals (μm) using the Image Pro Plus software (Media Cybernetics). By comparing the images from 0 h to the last time point, the distances of each scratch were obtained.

### Western Blot Analysis

For Western blotting analysis, protein extraction from cultured NIH3T3 cells was conducted according to previously described methods (Xi et al., 2013; Chen et al., 2016). Whole cell lysates were separated using SDS/10% PAGE and electrophoretically transferred to a polyvinylidene fluoride membrane by standard procedures. After the membrane was blocked, primary antibodies were added to the membranes followed by incubation at 4°C for 12 h. After three washes in TBST (50 mM Tris, 150 mM NaCl, 0.1% Tween 20, pH 7.4), the membranes were incubated with the secondary antibody at 37°C for 1 h. Primary antibodies were raised against the specific rabbit anti-β-catenin primary polyclonal antibody (1:500 dilution; Cayman), rabbit anti-COX-2 primary polyclonal antibody (1:500 dilution; Sigma) and mouse anti-β-actin (1:1000 dilution; Abcam, Cambridge, MA, United States). Horseradish peroxidase-conjugated goat anti-rabbit or mouse IgG (3:10000; Boster, Wuhan, China) was used as the secondary antibody. Western blot analysis was performed as described above and immunoreactive protein bands were visualized using Beyoecl Plus according to the manufacturer’s instructions (Boster, Wuhan, China).

### Measurement of Cytokine Levels by ELISA

Prostaglandin E_2_ in cell-culture supernatants and cell were quantified by commercial ELISA kits (Bioss Antibodies) in accordance with the manufacturer’s instructions.

### Real-Time PCR Assay

Total RNA was extracted from cultured NIH3T3 cells using RNAiso Plus (TaKaRa Bio, Dalian, Liaoning, China), followed by cDNA synthesis according to the manufacturer’s instructions with the Advantage^®^ RT-for-PCR Kit (TaKaRa Bio, Dalian, Liaoning, China; Li et al., 2015). Thereafter, 2 μg of cDNA samples was used immediately to quantify the mRNA levels of *β-catenin, COX-2, TGF-β1*, and *HMGB-1* with the Thermal Cycler Dice TP800 system (TaKaRa Bio, Otsu, Shiga Prefecture, Japan) based on the SYBR Premix Ex Taq II (TaKaRa Bio, Dalian, Liaoning, China) with 30–40 cycles of denaturation at 95°C for 5 s, annealing and extension at 60°C for 30 s. Furthermore, the mRNA expression levels of the target genes were normalized to that of *GAPDH* where the sequences of both forward and reverse primers are listed in Table 1.

**Table 1:** Summary of the primers designed and used in the present work.

Gene	Direction	Primers for mouse (5′–3′)	PCR product (bp)
*β-Catenin*	F	CCTAGCTGGTGGACTGCAGAA	137
	R	CACCACTGGCCAGAATGATGA	
*COX-2*	F	CTGGAACATGGACTCACTCAGTTTG	109
	R	AGGCCTTTGCCACTGCTTGTA	
*TGF-β1*	F	GTGTGGAGCAACATGTGGAACTCTA	174
	R	CGCTGAATCGAAAGCCCTGTA	
*HMGB-1*	F	GACCCCAATGCACCCAAGAG	113
	R	TTCTTTGCAACATCACCAATGG	
*GAPDH*	F	TGTGTCCGTCGTGGATCTGA	150
	R	TTGCTGTTGAAGTCGCAGGAG	

### Immunofluorescence Staining

The NIH3T3 cells were plated on μ-slides with eight wells and incubated with PGE_2_, LPS, PGE_2_ (1 μg/mL)+AH6809 (1 μg/mL), or LPS (400 ng/mL)+AH6809 (1 μg/mL) for 24 h. Immunofluorescence staining was performed according to previously described methods (Zhang et al., 2017). The slides were gently washed twice with PBS. Subsequently, 4% paraformaldehyde was applied onto the PLL-coated slides prepared above for 30 min to adhere cells onto the slide adequately. Following a rinsing step, 0.5% Triton-X100 solution was applied to permeabilize cell membranes for 15 min. To enhance permeability and block non-specific antibody binding, samples were incubated in 1% BSA solubilizing in PBS (pH 7.4) for 1 h. Next, the protoplasts were incubated with 125 μl primary polyclonal antibody (rabbit anti-β-catenin, 1:200, Cayman; goat anti-COX-2, 1:200, Abcam) over night at 4°C in a box with moisture. Then, samples were incubated with 125 μl secondary antibody (Goat anti-Rabbit TRITC-conjugated, CWBIO, 1:100; Rabbit anti-Goat FITC-conjugated, Invitrogen, 1:500) for 1 h at the room temperature. Nuclear staining was performed with DAPI (Electron Microscopy Sciences, United States). Finally, the immunofluorescence quantification for β-catenin was performed by an inverted fluorescence microscope (Eclipse Ti, Nikon, Japan) with the use of image analysis software (NIS-Elements AR 3.0, Nikon) according to previously described method (Li et al., 2012).

### Luciferase Assay

It has been previously reported that COX-2 promoter contained a functional TCF/LEF-response element for the enhancement of β-catenin signaling (Nunez et al., 2011). Here, we assessed whether β-catenin contributed to the transcriptional regulation of the *COX-2* expression. For this purpose, core promoter region of *COX-2* gene, which contained the β-catenin binding site or mutated site was cloned into pGL3 luciferase reporter vector, then transfected into HEK293T cells (Dordelmann et al., 2008). Dual luciferase reporter assay was performed according to the manufacturer’s protocol (Promega). For promoter analysis, sequence of *COX-2* promoter, or the corresponding mutated sequences were cloned into the pGL3 promoter reporter vector and the reference vector pRL-SV40 were co-transfected with pcDNA3-mCtnnb1 into HEK293T cells. After transfection, lysed cell fractions were collected for luciferase measurement. Firefly luciferase activities were normalized by renilla luciferase activities. For plasmid transfection, cells were seeded into 24-well plates (Corning Inc., Corning, NY, United States) without antibiotics. After 24 h, transfections were carried out using Lipofectamine 2000 reagent (Invitrogen, Carlsbad, CA, United States) according to the manufacturer’s instructions. The efficiency of transfection was evaluated by fluorescence intensity using inverted fluorescence microscopy (Nikon, Japan).

### Statistical Analysis

All data were expressed as mean ± standard deviation (SD) and analyzed by one way analysis of variance (ANOVA) with Tukey test to determine group differences. Difference between two groups was compared by unpaired Student’s *t*-test. The analyses were performed using InStat software (GraphPad Software Inc., La Jolla, CA, United States). *P* < 0.05 was considered to be statistically significant.

## Results

### LPS Activated the Migratory Potential of NIH3T3 Cells

**FIGURE 1:**
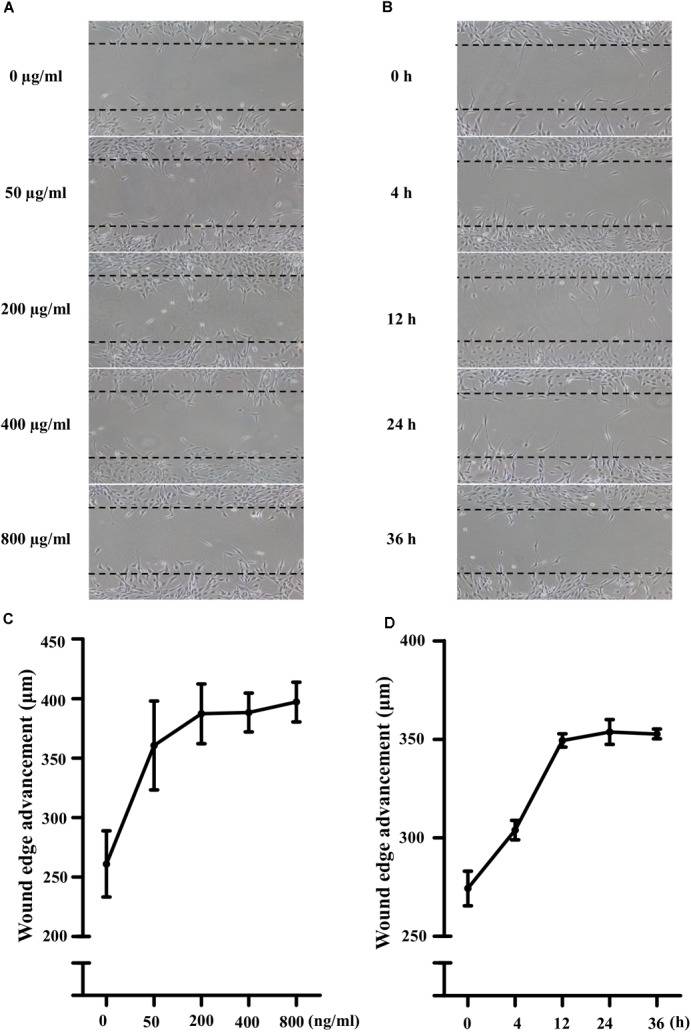
Lipopolysaccharide (LPS) activated the migration potential of NIH3T3 cells in dose- and time-dependent manners. **(A,C)** Treatment of NIH3T3 cells with LPS (0, 50, 200, 400, and 800 ng/ml) for 24 h activated migration of cells in a dose-dependent manner. **(B,D)** Treatment of NIH3T3 cells with 400 ng/ml LPS in the inductive period (0, 4, 12, 24, and 36 h). The wound edge advancement was counted by the photomicrographs under microscope and the results were measured using Image Pro Plus software. All assays were performed at least three times.

Lipopolysaccharide (50, 200, 400, and 800 ng/mL) did not affect the viability of NIH3T3 cells significantly. As shown in Figures 1A,C, compared with the control, LPS treatment increased the cell migration in a concentration-dependent manner. 400 ng/ml LPS increased the cells migration significantly (149 ± 6% of control, *P* < 0.05). Furthermore, to investigate the time-effect relationship, the scratch assay was conducted with 400 ng/ml LPS. Photographs of cellular migrations were taken at 4, 12, 24, and 36 h after scratching. Representative photomicrographs were shown in Figures 1B,D. Compared with the control, LPS treatment increased the cell migration in a time-dependent manner (4 h, 111 ± 2% of control; 24 h, 129 ± 2% of control, *P* < 0.01; 36 h, 129 ± 1% of control, *P* < 0.01). Thus, 400 ng/ml LPS treatment for 24 h was used for further scratch assay.

### LPS Increased Expressions of Both COX-2 and β-Catenin in NIH3T3 Cells

**FIGURE 2:**
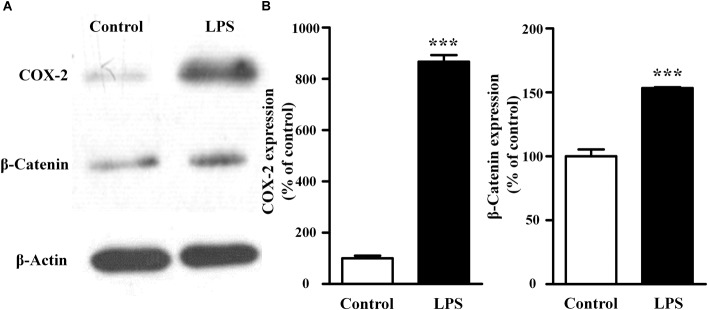
Effects of LPS on the endogenous expression of COX-2 and β-catenin in NIH3T3 cells. Representative blots **(A)** and summary **(B)** of the COX-2 and β-catenin expression in NIH3T3 cells treated with or without LPS (400 ng/ml, 24 h); ^∗∗∗^*P* < 0.001 vs. control, *n* = 5.

As shown in Figure 2, compared with the control group, LPS treatment increased both COX-2 and β-catenin expressions (867 ± 37% of the control for COX-2, *P* < 0.001; 153 ± 1% of the control for β-catenin, *P* < 0.001). These results suggested that the activation of fibroblast migration by LPS was associated with increases of COX-2 and β-catenin expressions in fibroblasts.

### COX-2 and β-Catenin Contributed to the LPS-Induced NIH3T3 Cell Migration

**FIGURE 3:**
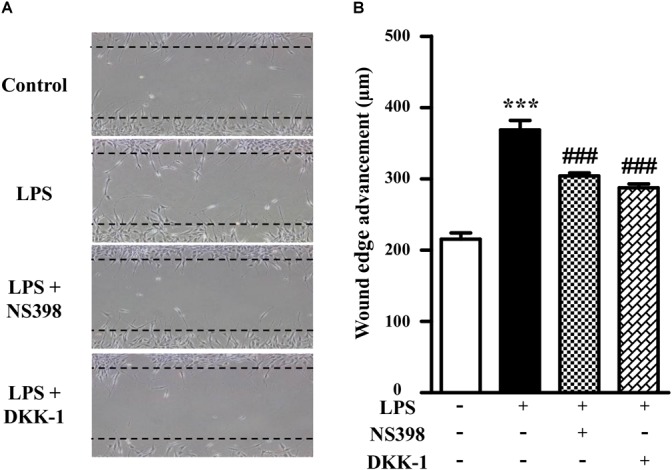
β-Catenin interacted with COX-2 in the NIH3T3 cells treated with LPS. **(A)** Treatment of NIH3T3 cells with NS398 or DKK-1 for 24 h inhibited the cell migration potential. The wound edge advancement was counted by Image Pro Plus software. **(B)** Quantification of the scar widths. ^∗^*P* < 0.05, ^∗∗^*P* < 0.01, and ^∗∗∗^*P* < 0.001 vs. control; ^#^*P* < 0.05 and ^###^*P* < 0.001 vs. the LPS group, *n* = 5.

**FIGURE 4:**
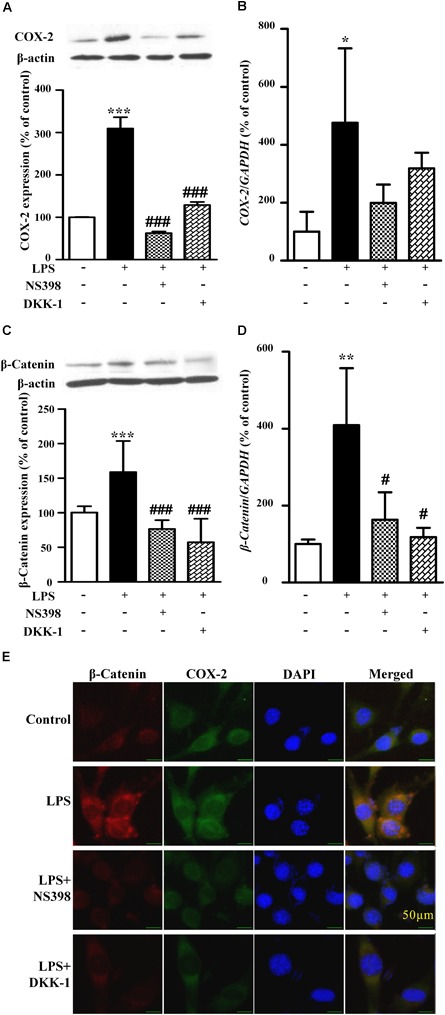
The protein expression and mRNA levels of β-catenin and COX-2 in the NIH3T3 cells treated with LPS. The protein and mRNA expression quantitative analysis of COX-2 **(A,B)** or β-catenin **(C,D)** in NIH3T3 cells. The quantitative analysis of protein was measured by ImageJ. ^∗^*P* < 0.05, ^∗∗^*P* < 0.01, and ^∗∗∗^*P* < 0.001 vs. control; ^#^*P* < 0.05 and ^###^*P* < 0.001 vs. the LPS group, *n* = 3. **(E)** The immunofluorescence staining of β-catenin and COX-2 in the NIH3T3. Scale bars: 50 μm.

As shown in Figure 3, compared to the LPS-treated group (171 ± 6% of control), NS398 or DKK-1 significantly inhibited cellular migrations (141 ± 2%, *P* < 0.001; 133 ± 3%, *P* < 0.001, respectively). LPS induced the mRNA and protein levels of COX-2 and β-catenin (Figures 4A–D). However, NS398 or DKK-1 could reverse that impact of LPS (Figures 4A–D). These data suggested that COX-2 and β-catenin contributed to the LPS-induced migration of NIH3T3 cells.

### COX-2 Was Upregulated by Wnt/β-Catenin Pathway

**FIGURE 5:**
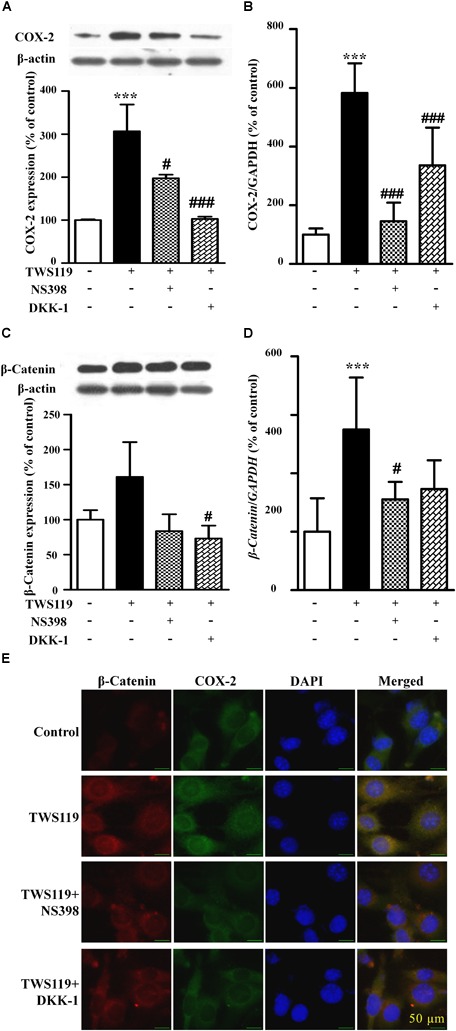
The protein expression and mRNA levels of β-catenin and COX-2 in the NIH3T3 cells treated with TWS119. The protein expression and mRNA quantitative analysis of COX-2 **(A,B)** or β-catenin **(C,D)** in NIH3T3 cells. ^∗^*P* < 0.05, ^∗∗^*P* < 0.01, and ^∗∗∗^*P* < 0.001 vs. control; ^#^*P* < 0.05 and ^###^*P* < 0.001 vs. the LPS group, *n* = 3. **(E)** The immunofluorescence staining of β-catenin and COX-2 in the NIH3T3 cells. Scale bars: 50 μm.

As shown in Figures 5A,B, compared with the control, TWS119 increased both the protein and mRNA expressions of COX-2 (161 ± 50% of control, *P* < 0.0001; 583 ± 101% of control, *P* < 0.0001). Furthermore, the protein and mRNA expressions of COX-2 in the TWS119 + NS398 treated group were significantly decreased in comparison with that of the TWS119 treated group. There was a same trend in the TWS119 + DKK-1 treated group. As shown in Figures 4E, 5E, immunofluorescence staining demonstrated that COX-2 and β-catenin (Figures 5C,D) were mainly distributed in the cytoplasm of the cells. The upregulation of COX-2 and β-catenin was correlated significantly with the treatment of LPS or TWS119, while their expressions were inhibited significantly by DKK-1 or NS398. Those results were in accordance with Western blot.

### PGE_2_/EP2 Was Involved in LPS-Induced β-Catenin Nuclear Translocation

**FIGURE 6:**
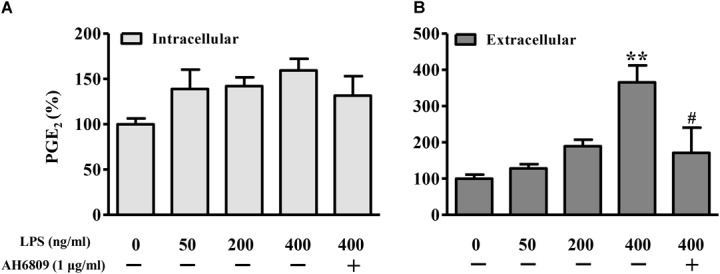
Lipopolysaccharide induced PGE_2_ production in NIH3T3 cells. The NIH3T3 cells were plated on plated on 35-mm dishes and incubated with LPS or AH6809 for 24 h. Both **(A)** intracellular and **(B)** extracellular PGE_2_ concentrations were detected using ELISA method. ^∗^ *P* < 0.05, ^∗∗^*P* < 0.01 vs. control; ^#^*P* < 0.05 vs. the LPS (400 ng/mL) group, *n* = 5.

**FIGURE 7:**
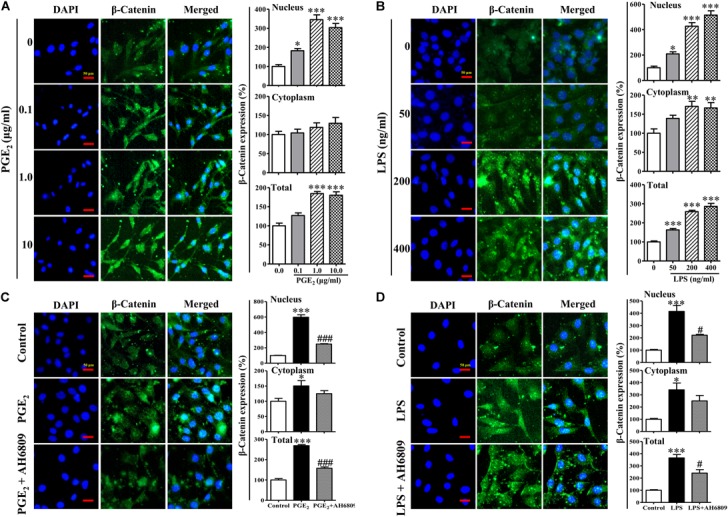
The nuclear translocation of β-catenin induced by prostaglandins E_2_ (PGE_2_) and LPS. The NIH3T3 cells were plated on μ-slides with eight wells and incubated with PGE_2_ **(A)**, LPS **(B)**, PGE_2_ (1 μg/mL)+AH6809 (1 μg/mL) **(C)**, or LPS (400 ng/mL)+AH6809 (1 μg/mL) **(D)** for 24 h. Fluorescence of the β-catenin expression was quantified by image analysis software (NIS-Elements AR 3.0, Nikon). Scale bars: 50 μm. ^∗^*P* < 0.05, ^∗∗^*P* < 0.01, and ^∗∗∗^*P* < 0.001 vs. control; ^#^*P* < 0.05 vs the LPS group in **(D)**; ^###^*P* < 0.001 vs the PGE2 group in **(C)**, *n* = 3.

Lipopolysaccharide increased the PGE_2_ production in a concentration-dependent manner (Figure 6). As shown in Figures 7A,B, both PGE_2_, and LPS increased the expression and nuclear translocation of β-catenin in dose-dependent manners. AH6908, an antagonist of PGE_2_ receptor 2 (EP2), alleviated the effects of PGE_2_, or LPS (Figures 7C,D). These data suggested that PGE_2_/EP2 was involved in LPS-induced β-catenin nuclear translocation.

### Both LPS and TWS119 Activated the mRNA Expression of *TGF-β1* and *HMGB-1*

**FIGURE 8:**
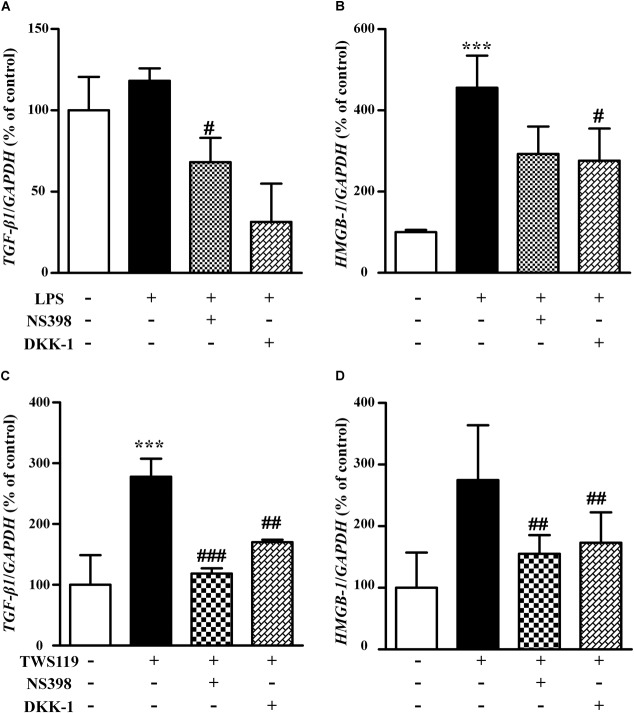
The mRNA levels and quantitative analysis of *TGF-β1* and *HMGB-1* in the NIH3T3 cells treated with LPS or TWS119. The mRNA levels and quantitative analysis of *TGF-β1* **(A)** or *HMGB-1* **(B)** in NIH3T3 cells treated with or without LPS plus NS398 or LPS plus DKK-1. The mRNA levels and quantitative analysis of *TGF-β1* **(C)** or *HMGB-1* **(D)** in NIH3T3 cells treated with or without TWS119 plus NS398 or TWS1119 plus DKK-1. ^∗∗∗^*P* < 0.001 vs. the control; ^#^*P* < 0.05, ^##^*P* < 0.01, and ^###^*P* < 0.001 vs. the LPS group, respectively.

To explore potential mechanisms for the stimulatory effects of LPS and TWS119 on NIH3T3 cells, we assessed the mRNA expression of *TGF-β1* and *HMGB-1*. As shown in Figure 8, the mRNA expression of *TGF-β1* and *HMGB-1* was increased after LPS or TWS119 treatment. And the addition of NS398 or DKK-1 attenuated those impacts.

### COX-2 Expression Was Activated by β-Catenin

**FIGURE 9:**
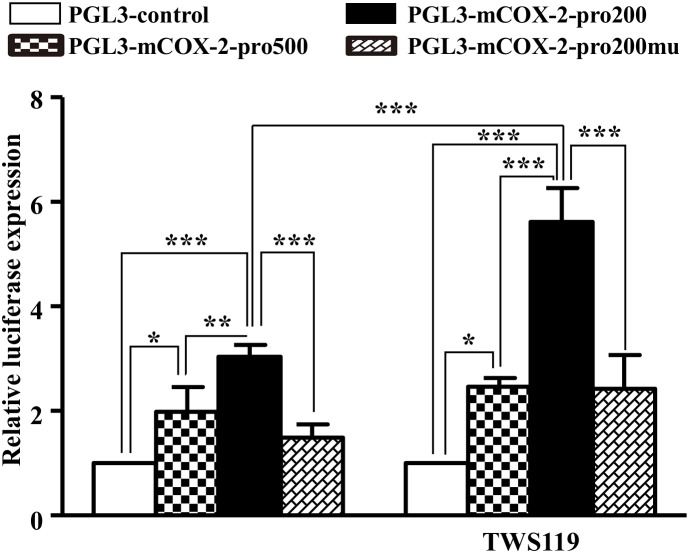
β-Catenin regulated promoter activity of COX-2. The promoter activity of COX-2 was measured by the luciferase reporter assay. The core promoter region containing β-catenin binding site or the mutant site which could not bind by β-catenin was cloned into luciferase reporter vector. After transfection into HEK293T cells, luciferase activities were measured with or without TWS119 addition; ^∗^*P* < 0.05, ^∗∗^*P* < 0.01, ^∗∗∗^*P* < 0.001.

An obviously increased luciferase activity was observed in the *COX-2* promoter with upregulation of β-catenin indirectly by TWS119 (Figure 9). Comparing to the PGL3-control, the luciferase expression of *COX-2* (pro500, pro200, and pro200mu) was 1.98 ± 0.48, *P* < 0.05; 3.04 ± 0.23, *P* < 0.0001; 1.49 ± 0.25, respectively. TWS119 increased the luciferase expression of PGL3-mCOX-2-pro200 significantly, but not control, pro500, or pro200mu.

## Discussion

Wound healing assays are often studied as a model system for *in vivo* wound repair, where fibroblasts play an important role in forming the granulation tissue. In accordance with veritable scar formation, the scratch assay functions are as an appropriate model to mimic the process of fibroblast migrations under the condition of destroyed skin (Goetsch and Niesler, 2011). In wound healing assays, presence of free space, loss of cell-cell contacts, cell debris resulting from cell destruction, all play a role in the cellular response resulting in wound closure. Following a wound, inflammatory pathways activated by extracellular stimuli have all been shown to be involved in the response in fibroblasts. Along these pathways, how β-catenin/COX-2 contribute to fibroblasts migration remains poorly understood.

Since migration of fibroblasts was a major process of wound healing, we first checked the effects of LPS on NIH3T3 cells using the *in vitro* scratch assay. LPS treatment increased the cell migration in concentration-and time-dependent manner. To evaluate whether the LPS-induced migration of NIH3T3 cells was associated with COX-2 and β-catenin, we used inhibitors. And NS398, a COX-2 inhibitor, inhibited LPS-induced NIH3T3 cells migration. DKK-1, an antagonist of the Wnt/β-catenin signaling, also inhibited that migration. However, TWS119, an inducer of β-catenin *via* GSK-3β, increased the cell migration. We further quantified the protein expressions of COX-2 and β-catenin using RT-PCR, Western blot, and immunofluorescence staining methods. LPS or TWS119 treatment increased COX-2 and β-catenin expressions, and that could be attenuated by NS398 or DKK-1 addition. There is a classic signal pathway between COX-2 and β-catenin in cancer cell migration: COX-2 increases the PGE_2_ production, and PGE_2_ binds to EP2. This leads to the inactivation of GSK-3β, thereby relieving the inhibitory phosphorylation of β-catenin and activating its signaling pathway (Castellone et al., 2005; Singh and Katiyar, 2013). In the present study, we imported that COX-2/β-catenin pathway into LPS-induced migration of NIH3T3 cells. All evidence indicated that it worked very well. LPS induced the PGE_2_ production, and PGE_2_ increased the expression and nuclear translocation of β-catenin, while EP2 blocker alleviated those effects.

*TGF-β1* and *HMGB-1* are important regulators of both inflammation and fibroblasts migration (Wang et al., 2011; Pandolfi et al., 2016; Bianchi et al., 2017). Our results showed that *TGF-β1* and *HMGB-1* mRNA were increased by LPS or TWS119, and reduced by COX-2/β-catenin inhibitor. Thus, *TGF-β1* and *HMGB-1* might be the downstream of COX-2/β-catenin pathway and contribute to the LPS-induced migration of NIH3T3 cells. As a target of the β-catenin signaling pathway, *HMGB-1* could directly bind to the promoter regions and subsequently activate the transcription of COX-2 in human umbilical vein endothelial cells (Ji et al., 1998) and pancreatic epithelial cells (Hillion et al., 2012), suggesting the existence of an positive feedback pathway that could lead to the up-regulation of COX-2 expressions by β-catenin.

Moreover, DKK-1, an antagonist of the Wnt/β-catenin signaling, abolishes the expressions of β-catenin proteins and translocations induced by LPS or TWS119. That further revealed the possibility of a positive feedback between COX-2 and β-catenin. This crosstalk between COX-2 and β-catenin could be explained by the COX-2 promoter containing a functional TCF/LEF-response element for the enhancement of β-catenin signaling (Nunez et al., 2011). Furthermore, the luciferase assay confirmed the crosstalk between COX-2 and β-catenin. When NS398, DKK1, or TWS119 used in the current study, a very common and interesting phenomenon in pharmacology was observed: NS398 downregulated COX-2 expression; DKK1 downregulated β-catenin expression; TWS119 upregulated β-catenin expression. There have been plenty of studies which are consistent with our current results (Du et al., 2008; Li et al., 2015; Ye et al., 2016). As a selective inhibitor of COX-2 bioactivity, NS-398 is able to mitigate the COX-2-mediated production of downstream prostaglandins and the subsequent inflammatory response, including COX-2 expression. That is a positive feedback. The same mechanism also works in TWS119-induced β-catenin expression.

In order to investigate the pathway, we indeed used inhibitors. These inhibitors are carefully selected and we have checked their action points, and the inhibitor controls were set for every experiment (Supplementary Material). However, transfection transcriptional activation would be more evident than inhibitor application.

## Conclusion

**FIGURE 10:**
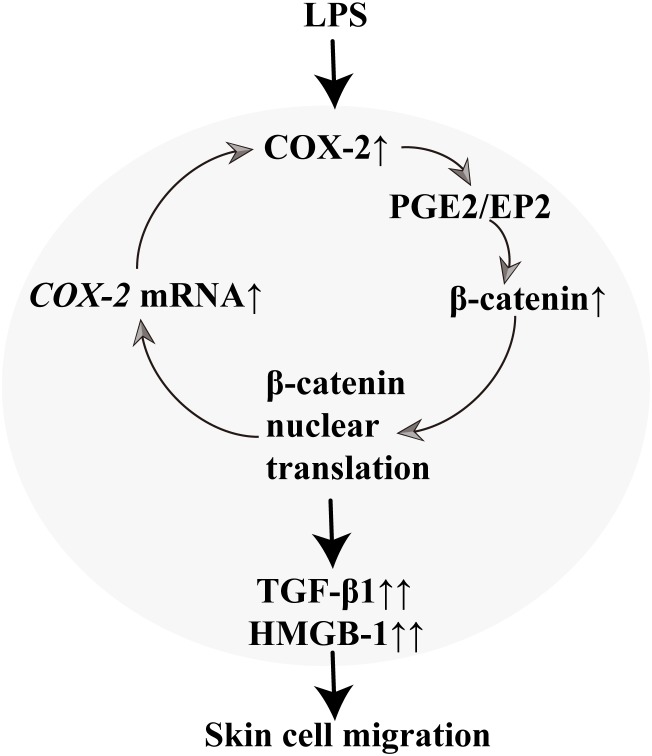
The scheme of the β-catenin and COX-2 crosstalk.

Within this work, wound healing assays, performed on fibroblast monolayers, revealed an interesting crosstalk between β-catenin and COX-2. LPS stimulated the NIH3T3 fibroblasts migration through a positive feedback between β-catenin and COX-2, in which PGE_2_, EP2, *TGF-β1*, and *HMGB-1* played as signal molecules (Figure 10).

## Author Contributions

H-BT conceived and designed the experiments. X-JL, F-ZH, YW, YX, and H-BT performed the experiments. H-BT, F-ZH, Y-SL, X-JL, WZ, YX, and G-HT analyzed the data and wrote the paper. H-BT, F-ZH, and YW contributed reagents, materials, and analysis tools.

## Conflict of Interest Statement

The authors declare that the research was conducted in the absence of any commercial or financial relationships that could be construed as a potential conflict of interest.
