# Supplementary material for: Lipopolysaccharide Stimulated the Migration of NIH3T3 Cells Through a Positive Feedback Between β-Catenin and COX-2
Source: Front Pharmacol. 2018 Dec 19;9:1487. doi: 10.3389/fphar.2018.01487 (PMC6305731; doi:10.3389/fphar.2018.01487)
Supplement: Supplementary file 1 [file Image_1.PDF]

frontiers

Supplementary Material

Lipopolysaccharide stimulated the migration of NIH3T3 cells through the crosstalk between β-catenin and COX-2

Feng-Zhen Huang[1,#], Xiao-Jun Li[1,#], Yan Wan[1], Yu-Sang Li[1], Wei Kevin Zhang[1], Yang Xi[2,*],

Gui-Hua Tian[3,*], He-Bin Tang[1,3,4,*]

***Corresponding authors:**

Tel/Fax: +86 27 6784 2332; E-mail: hbtang2006@mail.scuec.edu.cn (Tang HB).

Tel/Fax: +86 574 8760 0754; E-mail: xiyang@nbu.edu.cn (Xi Y);

Tel: +86 13717697488; E-mail: rosetgh@163.com (Tian GH)

1. Cell culture

NIH3T3 cells were obtained from the China Center for Type Culture Collection (CCTCC, Wuhan, China). The cells were cultured in high-glucose Dulbecco's modified Eagle's medium (DMEM) at 37˚C in a humidified atmosphere of 5% CO_2. The media were supplemented with 10% heat-inactivated calf serum and 1% penicillin/streptomycin. The cells were plated on 35-mm dishes.

2. Immunofluorescence staining

Immunofluorescence staining was performed according to previously described methods (Zhang et al., 2017). The slides were gently washed twice with PBS. Subsequently, 4% paraformaldehyde was applied onto the PLL-coated slides prepared above for 30 min to adhere cells onto the slide adequately. Following a rinsing step, 0.5% Triton-X100 solution was applied to permeabilize cell membranes for 15 min. To enhance permeability and block non-specific antibody binding, samples were incubated in 1% BSA solubilizing in PBS (pH 7.4) for 1 h. Next, the protoplasts were incubated with 125 μl primary polyclonal antibody (rabbit anti-β-catenin, 1:200, Cayman; goat anti-COX-2, 1:200, Abcam) over night at 4°C in a box with moisture. Then, samples were incubated with 125 μl secondary antibody (Goat anti-Rabbit TRITC-conjugated, CWBIO, 1:100; Rabbit anti-Goat FITC-conjugated, Invitrogen, 1:500) for 1 h at the room temperature. Nuclear staining was performed with

DAPI (Electron Microscopy Sciences USA). Images were captured with a digital camera coupled with a fluorescent microscope (Nikon, Japan).

Zhang, W.K., Gu, H.W., Li, X.J., Li, Y.S., Tang, H.B., Tian, G.H., et al. (2017). The dark side of "the force" - lipid nanoparticles enhance the oncogenesis of diethylnitrosamine and result in liver cancer in mice. *Nanomedicine* 13(2), 701-711. doi: 10.1016/j.nano.2016.09.017.

3. Supplementary Figure

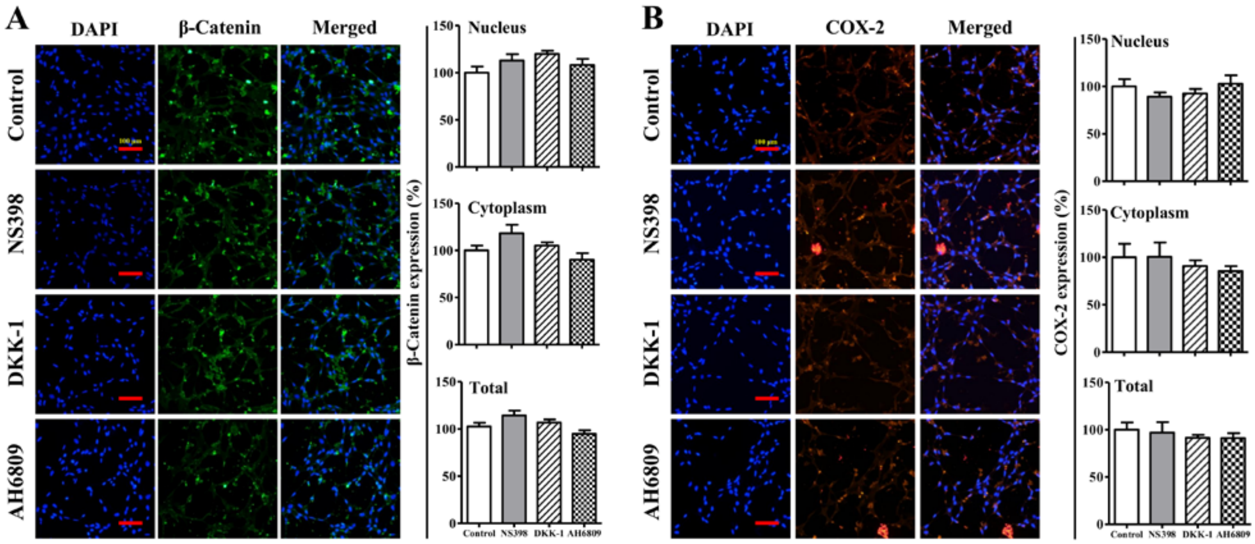

Supplementary Figure 1. Effects of NS398, DKK-1 and AH6809 used alone on the expression of β-catenin and COX-2. The NIH3T3 cells were plated on 35 mm dishes and incubated with NS398 (10 µM), DKK-1 (100 ng/mL), or AH6809 (1 µg/mL) for 24 h. The green or red fluorescence indicated positive expression of β-catenin or COX-2. The blue indicated the nuclei stained with DAPI under the same field (×40). Scale bars: 100 µm..
